# Cor Triatriatum Sinister With a Decompressing Levoatriocardinal Vein: A Rare Association

**DOI:** 10.7759/cureus.66610

**Published:** 2024-08-10

**Authors:** Yasser A Bhat, Abdulkader M Alsharif, Osama Alrusayni, Mohammed A Rashed, Abdulrahman Almesned, Abdullah Al Qwaee

**Affiliations:** 1 Pediatric Cardiology, Prince Sultan Cardiac Center, Buraidah, SAU

**Keywords:** neonate, surgical emergencies, severe pulmonary arterial hypertension, levoatriocardinal vein, cor triatriatum sinister

## Abstract

Levoatriocardinal vein has rarely been described with cor triatriatum. We report a case of a newborn with respiratory distress who was found to have cor triatriatum sinister with a decompressing levoatriocardinal vein on transthoracic echocardiography. The pulmonary venous confluence received all pulmonary veins and drained to the proximal left atrial chamber through significant communication. Moreover, the cor triatriatum membrane separated the left atrial body into proximal and distal left atrial chambers with a restrictive opening in the membrane, causing severe flow limitation to the cardiac output, severe pulmonary arterial hypertension, and significant right ventricular dilatation. In addition, a sizeable levoatriocardinal vein decompressed the pulmonary venous confluence drained superiorly to the left innominate vein. She had a successful surgical repair by resectioning the cor triatriatum membrane and ligating the levoatriocardinal vein. The patient had improved pulmonary arterial pressures and right ventricular dimensions at the one-month follow-up. The case highlights the rare association of the levoatriocardinal vein and cor triatriatum, and its presence signifies severe obstruction at the level of cor triatriatum.

## Introduction

Cor triatriatum sinister is a rare heart abnormality characterized by a fibromuscular membrane in the left atrium, thus forming a proximal chamber receiving the pulmonary veins and a distal chamber communicating with the mitral valve and the left atrial appendage. This membrane has one or more perforations. An atrial septal defect (ASD) is sometimes located between the proximal or distal chamber and the right atrium [1]. The levoatriocardinal vein (LACV) is an even rarer vascular anomaly that connects the left atrium (or pulmonary vein tributaries) with the left innominate vein (LIV). Development of the pulmonary veins and systemic venous sinus is spatiotemporal during embryogenesis [2]. The association of the LACV with cor triatriatum is rare and has only been reported in a few case reports. We report a neonate with cor triatriatum sinister who had a LACV to the LIV and severe pulmonary hypertension.

## Case presentation

**Figure 1:**
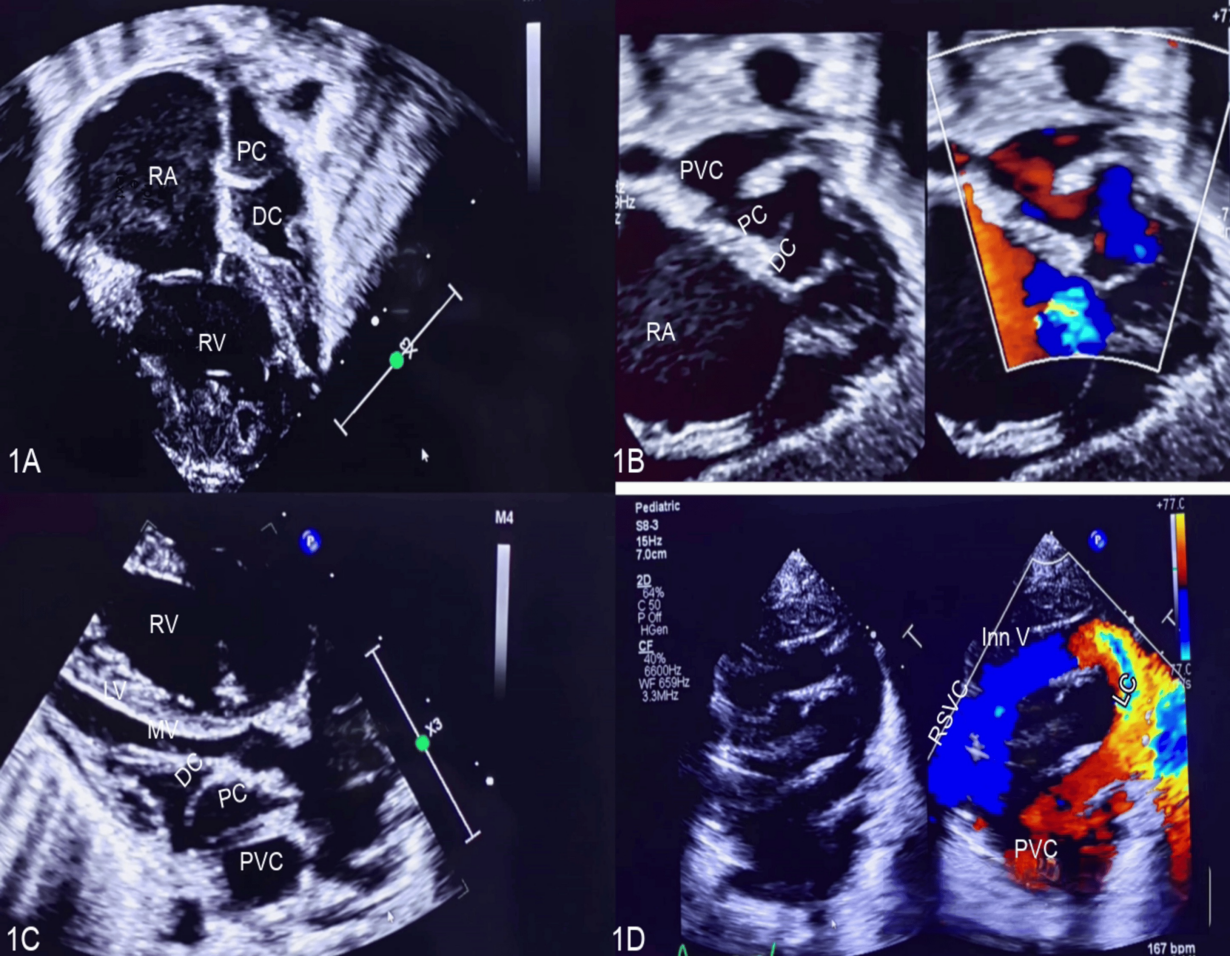
Transthoracic echocardiographic findings of the cor triatriatum and levoatriocardinal vein 1A: The four-chamber view shows that the left atrium is divided into the proximal chamber (PC) and distal chamber (DC) by a cor triatriatum membrane with a significant dilatation of the right atrium (RA) and right ventricle (RV). 1B: The colour compare subcostal image shows pulmonary venous confluence (PVC), proximal and distal chambers. IC: The parasternal long-axis view shows the squashed left ventricle due to pulmonary hypertension. 1D: The levoatriocardinal vein (LC) connects the pulmonary venous confluence to the left innominate vein (inn V). RSVC, right superior venacava

A four-day-old neonate was referred to the emergency room for respiratory distress. She was born full-term by normal vaginal delivery with a birth weight of 3 kg. Clinically, she had tachypnea, tachycardia, cardiac murmur, and hepatomegaly. Transthoracic echocardiography showed an intra-atrial membrane proximal to the left atrial appendage, separating the left atrium into proximal and distal chambers. The membrane had a tiny perforation, causing severe obstruction of the blood flow. Moreover, the proximal left atrial chamber communicated with the right atrium through a small atrial septal defect (ASD). The pulmonary venous confluence (PVC) is drained into the high-pressure proximal chamber via unrestricted communication. Additionally, LACV from PVC drained superiorly to LIV (Figures 1A-1D).

After stabilization, the patient had a successful surgical intervention in the form of resection of the intra-atrial membrane, levoatriocardinal vein ligation, and partial closure of the ASD.

Surgical technique and findings

After median sternotomy, thymectomy, and pericardial patch harvesting, the patent ductus arteriosus (PDA) was ligated. Then, under cardiopulmonary bypass, the vertical vein was dissected and isolated. The left atrium was accessed through a right atriotomy and the ASD. The rest of the interatrial septum was excised to expose the membrane. Subsequently, the thick circumferential membrane within the left atrium was excised. The ASD was closed using an autologous pericardial patch, and the levoatriocardinal vein was ligated. The patient was shifted to the intensive care unit (ICU) with an open sternum closed on the second postoperative day. The cardiopulmonary bypass and cross-clamp times were 50 and 40 minutes, respectively.

**Figure 2:**
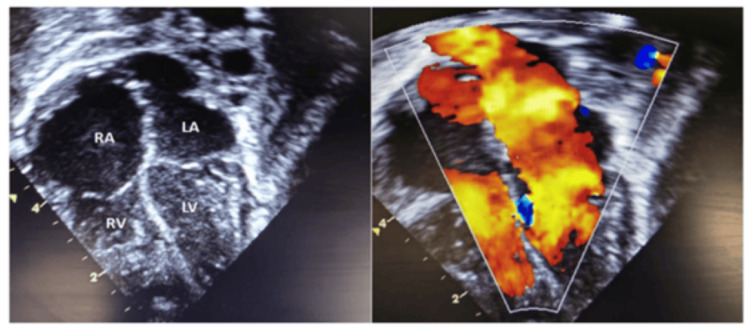
The post-operative colour compare image in a four-chamber view shows no residual cor triatriatum membrane with laminar flow from the pulmonary venous confluence to the left ventricle Moreover, dilatation of the right atrium (RA) and right ventricle (RV) improved significantly. LA, left atrium; LV, left ventricle

The patient developed a pulmonary hypertensive crisis in the ICU that responded to sedation, muscle relaxation, and inhaled nitric oxide. Later, the patient was extubated and weaned from inotropic support on the third postoperative day. The total duration of mechanical ventilation was 72 hours. In addition, ICU and hospital stays were six and 14 days, respectively. Follow-up echocardiography revealed an unobstructed flow from the PVC to the left ventricle. However, the patient had mild pulmonary hypertension, mild tricuspid regurgitation, and an estimated right ventricular systolic pressure of 45 mmHg (Figure 2).

## Discussion

Cor triatriatum may occur singly or in association with other congenital heart defects. The three principal embryological theories explaining the development of cor triatriatum are mal-septation, mal-incorporation, and entrapment of the common pulmonary vein. Typically, an ASD exists between the right atrium and the proximal or distal left atrial chambers [3]. Other associated abnormalities could include a left superior vena cava opening to the coronary sinus, ventricular septal defects, and anomalies of pulmonary venous drainage [1]. In cor triatriatum, a fibromuscular membrane divides the left atrium into a venous portion, called the proximal chamber, and a true left atrium portion, referred to as the distal chamber. In nearly all cases, the left atrial appendage is distal to the cor triatriatum membrane. [4] The levoatriocardinal vein is a rare embryonic vestige that flows out of a pulmonary vein or left atrium into the superior vena cava or innominate vein. It is generally associated with left atrial outflow obstruction, such as mitral stenosis or atresia and aortic stenosis or atresia. Moreover, it has been associated with cor triatriatum [5]. Only six reported cases of pediatric patients with concomitant cor triatriatum sinister and levoatriocardinal vein are in the literature [2].

Patients usually develop pulmonary edema in infancy unless a large opening in the membrane permits adequate drainage of the pulmonary veins. This opening may become obstructed later in life due to fibrosis and calcification and may cause symptoms to develop [6]. Diagnosis is usually based on echocardiography, which defines membrane characteristics and associated lesions. Additionally, transesophageal and three-dimensional echocardiography are useful tools for evaluating this condition. Furthermore, other imaging modalities include multi-slice computer tomography and magnetic resonance imaging. Surgical treatment is recommended in children and adults with symptoms of significant obstruction [7]. In the case of an isolated levoatriocardinal vein with a significant left to right shunt, the treatment options are surgical ligation or percutaneous placement of an Amplatzer occluder device [2]. Two cases of successful balloon catheter dilation of the communication between the proximal and distal chambers with follow-up periods of three and 12 months have been reported; however, the long-term outcome of balloon dilatation remains to be determined [8].

This case report emphasizes the association of the levoatriocardinal vein with cor triatriatum sinister, which has been described earlier in a few cases. Although rare, the presence of LACV must be considered when assessing patients with cor triatriatum, as it requires surgical ligation in addition to cor triatriatum repair.

## Conclusions

The presence of the levoatriocardinal vein, which is typically associated with mitral and aortic atresia, has been observed in rare cases of cor triatriatum sinister. In patients with cor triatriatum, it is crucial to address the levoatriocardinal vein during the corrective procedure to prevent the occurrence of a left-to-right shunt post-repair. This case report draws attention to the uncommon but significant association between cor triatriatum and the levoatriocardinal vein. It is worth noting that the presence of the levoatriocardinal vein may delay the onset of symptoms due to its decompressing effect. However, its existence also indicates a severe flow obstruction across the cor triatriatum membrane.
